# Inhibitory Effect of Delphinidin on Oxidative Stress Induced by H_2_O_2_ in HepG2 Cells

**DOI:** 10.1155/2020/4694760

**Published:** 2020-11-20

**Authors:** Jingjing Xu, Yanwei Zhang, Guofeng Ren, Rengui Yang, Jingfang Chen, Xiaojing Xiang, Hong Qin, Jihua Chen

**Affiliations:** ^1^Xiangya School of Public Health, Central South University, Changsha 410078, China; ^2^Hunan Provincial Key Laboratory of Clinical Epidemiology, Changsha 410078, China; ^3^Changsha Center for Disease Control and Prevention, Changsha 410002, China; ^4^Department of Neurology, Changsha Central Hospital, Changsha 410018, China

## Abstract

Chronic liver diseases (CLDs) are correlated with oxidative stress induced by the accumulation of intracellular reactive oxygen species (ROS). In this study, we employed HepG2, a human liver carcinoma cell line containing many antioxidant enzymes, to explore the function of delphinidin against oxidative stress induced by H_2_O_2_ and to provide scientific data of the molecular mechanism. Cells were pretreated with different concentrations of delphinidin (10 *μ*mol/L, 20 *μ*mol/L, and 40 *μ*mol/L) for 2 h before treatment with 750 *μ*M H_2_O_2_ for 1 h. The results showed that H_2_O_2_ decreased the survival rate of HepG2 cells and increased the level of ROS, but delphinidin pretreatment could possess the opposite result. At the same time, the expression of Nrf2 was enhanced by the delphinidin pretreatment. This was because delphinidin promoted Nrf2 nuclear translocation and inhibited its degradation, which led to the increase expression of antioxidant protein HO-1 (Nrf2-related phase II enzyme heme oxygenase-1). Besides, we found that delphinidin could significantly alleviate the reduction of Nrf2 protein levels and the accumulation of intracellular ROS levels in Nrf2 knockdown HepG2 cells. In conclusion, our study suggested that delphinidin, as an effective antioxidant, protected HepG2 cells from oxidative stress by regulating the expression of Nrf2/HO-1.

## 1. Introduction

The death toll of chronic liver disease (CLDs) associated with cirrhosis, liver cancer, hepatitis, etc. is on the rise worldwide [1]. Increasing evidences confirmed the contributory role of oxidative stress in the pathogenesis of CLDs. As the common cause of oxidative stress [2, 3], reactive oxygen species (ROS) are a collective name for molecules containing oxidative reactions, including superoxide anions (O_2_-), hydroxyl radicals (OH), hypochlorous acid (HOCl), ozone (O_3_), and hydrogen peroxide (H_2_O_2_) [4].

There is an adaptive and dynamic antioxidant defense system to eliminate excessive ROS and protect cells against oxidative stress in human body. Antioxidants, antioxidant enzymes, and phase II detoxifying enzymes are included in this system. Previous studies have shown that the phase II detoxification enzymes consisted of glutathione S-transferase (GST), NAD(P)H quinone oxidoreductase-1 (NQO1), and heme oxygenase-1 (HO-1) which could scavenge free radicals and electrophiles [5]. In addition, the antioxidant response element (ARE), a common nucleotide sequence in the promoter regions of phase II detoxifying enzymes, could be effectively activated by nuclear factor erythroid 2-related factor 2 (Nrf2) and could regulate the expression of its downstream genes [6].

As a key transcription factor, Nrf2 regulates the response of intracellular antioxidant and plays a crucial role in homeostasis maintenance [7]. Under normal conditions, Nrf2 is stored in the cytoplasm in an inactive state until it is coupled with Kelch-like ECH-associated protein 1 (Keap1) and rapidly degraded by the ubiquitin-protease system. Thereby the expression of Nrf2 is stable and the transcriptional activity is low at this time. While under oxidative stress conditions, Nrf2 is responsible for its separation with Keap1 and transfers into the nucleus before recognizing and binding with the ARE which regulates the transcription of downstream biphasic detoxification enzymes and antioxidant protein genes, such as GST, NQO1, and HO-1. Previous studies demonstrated that Nrf2 had protective effects against oxidative stress-induced diseases such as cancer [8], diabetes [9], respiratory diseases [10], chronic inflammation [11], cardiovascular diseases [12], and neurodegenerative diseases [13]. Other than that, several studies previously revealed that polyphenols activated the Nrf2/Keap1 pathway through different mechanisms to exert antioxidant activity such as quercetin [14, 15], epigallocatechin gallate [16], baicalein [17], and resveratrol [18].

Anthocyanins, which are widely distributed in edible plants, are a benefit to health [19–21]. For instance, anthocyanins have an effective function in neurological diseases, hypoglycemia, cardiotoxicity, and anti-inflammation [22–24]. Delphinidin, a natural anthocyanin, is one of the most valuable polyphenols due to its high antioxidant activity. Kim et al. previously revealed that delphinidin possessed antiangiogenic function potential [25]. Moreover, delphinidin could inhibit the proliferation of SKOV3 cells (ovarian cancer cells) [26] and it could prevent prostate cancer via inhibiting the apoptosis mediated by p53 [27]. Lee et al. reported that delphinidin could protect chondrocytes against H_2_O_2_-mediated oxidative stress by activating NF-*κ*B and Nrf2 [28]. However, there is a lack of data on how delphinidin regulates Nrf2 to exert the function of antioxidant stress. In this study, we intend to use H_2_O_2_ to construct a cell model of oxidative damage and evaluate the antioxidant activity of delphinidin. The purpose of this study is to investigate whether delphinidin exerts antioxidant protection on cells by regulating the Nrf2 pathway.

## 2. Materials and Methods

### 2.1. Cells and Reagents

HepG2 cells were obtained from Cancer Research Institute of Central South University (Changsha, China). Delphinidin was purchased from Cayman company (Michigan, USA). Hydrogen peroxide solution (H_2_O_2_) was purchased from HengXing Chemical Reagent (Tianjin, China). RPMI 1640 medium and fetal bovine serum (FBS) were purchased from Gibco (Grand Island, NY, USA). Penicillin–streptomycin solution and 3-(4,5-dimethylthiazol-2-yl)-2,5-diphenyltetrazolium bromide (MTT) reagent were obtained from Gen-View (Calimesa, CA, USA). The formation of intracellular ROS was determined using DCFH-DA (Sigma-Aldrich, St. Louis, MO, USA). The Nuclear and Cytoplasmic Protein Extraction Kit and protease inhibitor phenylmethylsulfonyl fluoride (PMSF) were bought from Beyotime (Shanghai, China). The primary antibodies, such as anti-Keap1, anti-HO-1, and anti-Lamin B were bought from Santa Cruz Biotechnology (CA, USA). The Nrf2 antibody was purchased from Abcam (Cambridge, UK). Antibodies against *β*-actin antibody and *α*-tubulin were obtained from ABclonal (Boston, MA, USA). Paraformaldehyde was purchased from Dingguo (Beijing, China). Triton-X was obtained from Solarbio (Beijing, China). Goat Serum and DAPI were purchased from Boster (Wuhan, China). Trizol reagent was obtained from Ambion (Austin, USA). qPCR SYBR Green Master Mix was bought from Vazyme (Nanjing, China). Reverse transcription kit instructions and Lipofectamine 3000 were purchased from Thermo Fisher Scientific (Wilmington, USA).

### 2.2. Cell Culture

HepG2 cells were cultured in RPMI 1640 medium, supplemented with 10% FBS and 1% penicillin–streptomycin solution at 37°C in a 5% CO_2_ atmosphere.

### 2.3. MTT Assay

MTT assay was performed to detect the effect of delphinidin on cell viability. HepG2 cells (~5 × 10^3^ cells/well) were seeded in 96-well plates for 24 h and then treated with different concentrations of delphinidin for 24 h. After changing the culture medium, 5 mg/mL MTT reagent was added and the cells were incubated for 4 h. Cell viability was calculated by measuring absorbance at 490 nm using a microplate reader. 10 *μ*M, 20 *μ*M, and 40 *μ*M delphinidin with the treatment of H_2_O_2_ were pretreated to assess the impact on cell viability based on the results of the above experiment.

### 2.4. ROS Assay

HepG2 cells (~5 × 10^3^ cells/well) were seeded in 96-well plates for 24 h. After refreshing the culture medium, cells were treated with delphinidin (10 *μ*mol/L, 20 *μ*mol/L, and 40 *μ*mol/L) for 2 h. And then the cells were exposed with H_2_O_2_ (750 *μ*mol/L) for 1 h. Cells were washed once with warm phosphate buffer saline (PBS) and a serum-free culture medium containing 5 *μ*M ROS probe was added and reacted for 30 min. The fluorescence was read with a spectrofluorimeter (PerkinElmer, Waltham, MA, USA) at excitation wavelength of 485 nm.

### 2.5. Western Blot Analysis

Cytoplasm and nuclear protein extraction was prepared by using the Nuclear and Cytoplasmic Protein Extraction Kit. Cells were washed 3 times with ice-cold 1×PBS and lysed in 140 *μ*L 1×SDS. Proteins were separated in a 10% sodium dodecyl sulfate-polyacrylamide gel electrophoresis (SDS-PAGE) and subsequently transferred to polyvinylidene difluoride (PVDF) membranes for 1.5 h before blocking in a 5% skimmed milk for 1 h. Then, the PVDF membranes were incubated into the specific primary antibody (Nrf2, Keap1, HO-1, *β*-actin, Lamin B, and *α*-tubulin) at 4°C overnight. After washing 3 times with Tris-Buffered Saline Tween20 (TBS-T) solution, the PVDF membranes were incubated with the corresponding secondary antibody at room temperature. Finally, the membranes were scanned in a chemiluminescence imaging system (Tanon 5500, Shanghai, China).

### 2.6. Immunofluorescence Assay

HepG2 cells (~3 × 10^4^ cells/well) were cultured for 24 h on coverslips in 24-well plates. Cells were treated with delphinidin (20 *μ*mol/L) for 3 h and then H_2_O_2_ (750 *μ*mol/L) for 1 h. The cells were subsequently washed twice with warm PBS and fixed with 4% paraformaldehyde at room temperature for 30 min. After permeation with 0.1% Triton-X at room temperature for 20 min, cells were blocked with Goat Serum for 1 h. Then, cells were incubated with Nrf2 primary antibody at 4°C overnight. After washing 3 times with cold PBS, cells were incubated with fluorescent secondary antibody for 1.5 h and stained with DAPI for 5 min in the dark at room temperature. Finally, coverslips were mounted onto glass slides, and the images were taken by fluorescence microscope (Thermo Scientific, Wilmington, USA).

### 2.7. Immunoprecipitation

HepG2 cells (~5 × 10^6^ cells/dish) were seeded in 10 cm petri dish for 24 h. Then, cells were treated with delphinidin (20 *μ*mol/L) for 2 h and H_2_O_2_ (750 *μ*mol/L) for 1 h. After washing twice with cold PBS, cells were lysed with cell lysis buffer containing 1 mM PMSF. The lysates were stirred for 1 h at 4°C and then centrifuged at 12,000 xg in a high-speed refrigerated centrifuge at 4°C for 15 min. After determining the protein concentrations of supernatants, the cell extracts were preincubated with 2 *μ*g normal rabbit immunoglobulin G (IgG) and 20 *μ*L Protein A agarose beads (Beyotime, Shanghai, China) for 2 h. The mixture was centrifuged at 2,500 rpm in a high-speed refrigerated centrifuge at 4°C for 5 min. The supernatants were added with anti-Nrf2 antibody (2 *μ*g) and stirred at 4°C overnight and then the supernatants were mixed with 30 *μ*L Protein A agarose beads for 2 h at 4°C. Subsequently, the mixture was centrifuged at 2,500 rpm in a high-speed refrigerated centrifuge at 4°C for 5 min to collect the beads. After washing once with cell lysis buffer, the beads were added into 1×SDS loading buffer and then heated at 100°C for 5 minutes for subsequent Western blotting experiments.

### 2.8. Quantitative Real-Time PCR

Total RNA was extracted from cells with trizol reagent, and 1% agarose gel was used to assess RNA integrity by using a UV gel imaging system. Total RNA was reverse transcribed into cDNA according to the reverse transcription kit instructions. Moreover, real-time PCR analysis was performed using a qPCR SYBR Green Master Mix and a LightCycler 96 Instrument (Roche, Basel, Switzerland). The primers used in the study were synthesized by Sangon Biotech (Sangon Biotech Co, Shanghai, China) and shown in Table 1. Finally, the data were calculated using the 2-*ΔΔ*Cq method.

### 2.9. Transfection of shRNA

The design of knockdown short hairpin ribonucleic acid (shRNA) (ccggacTGACAGAAGTTGACAATTActcgagTAATTGTCAACTTCTGTCAgttttt-tg) targeting Nrf2 was commissioned by GeneChem (Shanghai, China), and subsequently, the shRNA was cloned into double-marked lentiviral vector GV248 (GeneChem, Shanghai, China). Lipofectamine 3000 was used to transfect sh-con (negative control) and sh-Nrf2 plasmids into HepG2 cells. After puromycin treatment, nonspecific cells were eliminated and cells with green fluorescence were sorted using flow cytometry. Transfection cells were cultured and detected using Western blot and ROS methods.

### 2.10. Statistical Analysis

The SPSS18.0 statistical (Chicago, IL, USA) software was used for statistical analysis. All the data was repeated at least 3 times, and the results were expressed as the mean ± SD. The data was estimated the normality distribution and homogeneity of variance. Statistical differences between two groups were assessed using a two-tailed Student's *t*-test. The one-way analysis of variance was used between the multiple sample means (the pairwise comparison used the LSD test). Otherwise, the Kruskal-Wallis H test was used for data analysis (Student-Newman-Keuls test was used for pairwise comparison). Statistical significance was considered at *P* < 0.05.

## 3. Results

### 3.1. Delphinidin Protects HepG2 Cells against H_2_O_2_-Induced Oxidative Stress

**Figure 1:**
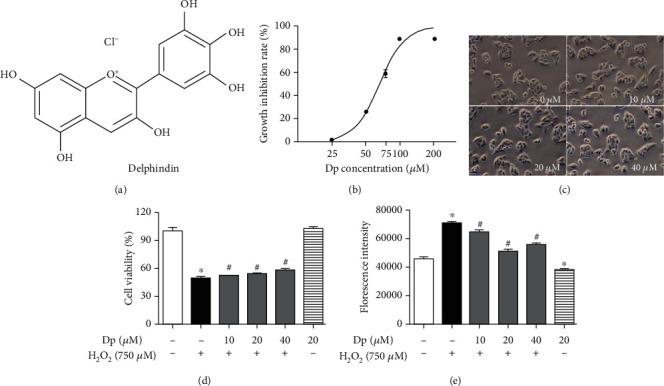
Effect of delphinidin on cell viability and the generation of ROS level induced by H_2_O_2_: (a) a chemical structure of delphinidin; (b) growth inhibition rate of HepG2 cells treated with different concentrations of delphinidin; (c) morphological observation of HepG2 cells treated with different concentrations of delphinidin; (d) cell viability of HepG2 cells treated with delphinidin and H_2_O_2_; (e) effects of delphinidin (10 *μ*M, 20 *μ*M, and 40 *μ*M) on intracellular ROS levels induced by H_2_O_2_. Values were presented as mean ± SD. ^∗^*P* < 0.05 vs. the control group and ^#^*P* < 0.05 vs. the H_2_O_2_-treated group.

HepG2 cells were treated with different concentrations (0~200 *μ*M) of delphinidin for 24 h to test the cytotoxicity, and the growth inhibition rate was measured by MTT assays. As shown in Figure 1(b), the IC50 value of HepG2 cells treated with delphinidin was 65.58 *μ*M. Compared with the control group, cells treated with delphinidin at different concentrations (10 *μ*M, 20 *μ*M, and 40 *μ*M) showed normal growth, polygonal shape, and well-defined boundaries (Figure 1(c)). According to the results of the above MTT experiments, 10 *μ*M, 20 *μ*M, and 40 *μ*M delphinidin were used to pretreat the cells and then the cell viability of HepG2 cells treated with H_2_O_2_ (750 *μ*M) was examined. On the one hand, the cell viability of the H_2_O_2_-treated group decreased significantly (100% and 50.84% ± 1.26%, *P* < 0.05). On the other hand, the delphinidin intervention group increased significantly. (50.84% ± 1.26% and 53.59% ± 0.51%, 55.58% ± 0.28%, and 59.52% ± 1.08%, respectively, *P* < 0.05) (Figure 1(d)).

Moreover, the 2′,7′-dichlorofluorescin diacetate (DCFH-DA) probe was used to detect intracellular ROS levels. Compared with the control group, the level of ROS in the H_2_O_2_-treated group increased by 60% (*P* < 0.05). On the contrary, compared with the H_2_O_2_-treated group, the level of ROS in the delphinidin intervention group (10 *μ*M, 20 *μ*M, and 40 *μ*M) decreased by 9.4%, 28.7%, and 21.1%, respectively (*P* < 0.05) (Figure 1(f)).

### 3.2. Activation of Nrf2-Related Proteins Regulated by Delphinidin

**Figure 2:**
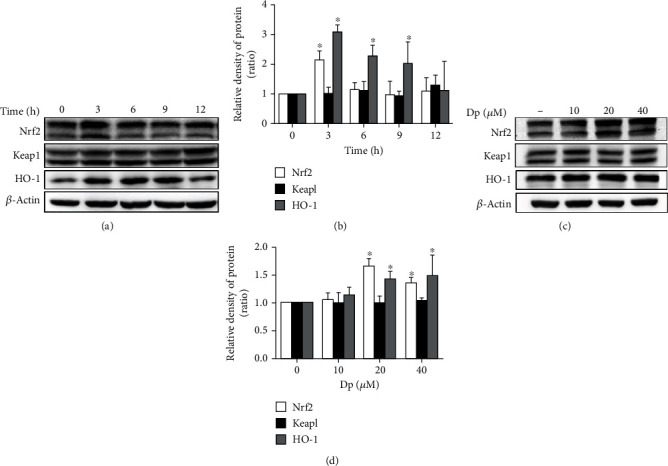
Nrf2-related protein expression was regulated by delphinidin in time and concentration dependence. The (a) protein bands and (b) relative protein expression of total Nrf2, Keap1, and HO-1 were reregulated by delphinidin (20 *μ*M) in time dependence. The (c) protein bands (d) and relative protein expression of total Nrf2, Keap1, and HO-1 were regulated by delphinidin (3 h) in different concentrations. *β*-Actin was used as a loading control for the total protein. Values were presented as mean ± SD. ^∗^*P* < 0.05 vs. the control group.

Compared with the control group, the protein expression of Nrf2 increased 1.2-fold at 3 h (*P* < 0.05) and that of HO-1 promoted at 3 h, 6 h, and 9 h (*P* < 0.05) after treatment with 20 *μ*M of HepG2 cells (Figures 2(a) and 2(b)). The results showed that cells treated with 20 *μ*M delphinidin had upregulated the level of Nrf2 and HO-1 protein at 3 h. Besides, the protein expression of Nrf2 and HO-1 was upregulated after 20 *μ*M and 40 *μ*M delphinidin treatment (*P* < 0.05). On the other hand, delphinidin did not affect the protein expression of Keap1 (Figures 2(c) and 2(d)). Therefore, 3 h processing time and 20 *μ*M delphinidin were chosen for the following experiment.

### 3.3. Activation of Nrf2/HO-1 Pathway by Delphinidin under Oxidative Stress

**Figure 3:**
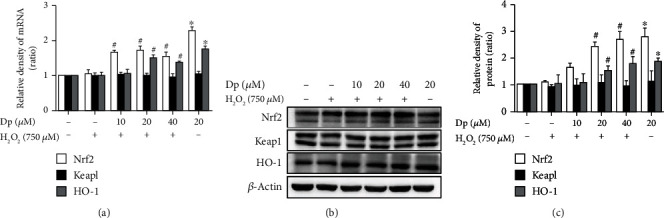
Delphinidin affected the level of Nrf2-related mRNA and protein in HepG2 cells under oxidative stress. (a) Effects of delphinidin (10 *μ*M, 20 *μ*M, and 40 *μ*M) and H_2_O_2_ (750 *μ*M) on Nrf2-related mRNA expression. The (b) protein bands and (c) relative protein expression of total Nrf2, Keap1, and HO-1 proteins were regulated by delphinidin (10 *μ*M, 20 *μ*M, and 40 *μ*M) under oxidative stress. *β*-Actin was used as a loading control for the total protein. Values were presented as mean ± SD. ^∗^*P* < 0.05 vs. the control group and ^#^*P* < 0.05 vs. the H_2_O_2_-treated group.

In order to investigate whether delphinidin could regulate the Nrf2/HO-1 pathway, HepG2 cells were treated with different concentrations (10 *μ*M, 20 *μ*M, and 40 *μ*M) of delphinidin for 2 h and subsequently treated with H_2_O_2_ (750 *μ*M) for 1 h (the total treatment time was 3 h). Then, the expression of Nrf2-related mRNA and protein was detected by qPCR and Western blot. Compared with the H_2_O_2_-treated group, the level of Nrf2 mRNA was significantly increased in delphinidin intervention groups (10 *μ*M, 20 *μ*M, and 40 *μ*M). Other than that, delphinidin resulted in a significant upregulation of HO-1 mRNA in 20 *μ*M and 40 *μ*M delphinidin groups. There was no significant difference in the expression of Keap1 mRNA (Figure 3(a)). As shown in Figures 3(b) and 3(c), we found that at concentrations of 20 *μ*M and 40 *μ*M also upregulated the protein expression of Nrf2 and HO-1. However, the level of Keap1 protein remained unchanged in each group (*P* > 0.05). This result showed that delphinidin could increase Nrf2 and HO-1 mRNA and protein levels, but had no effect on Keap1.

### 3.4. Delphinidin Promoted the Nuclear Translocation of Nrf2

**Figure 4:**
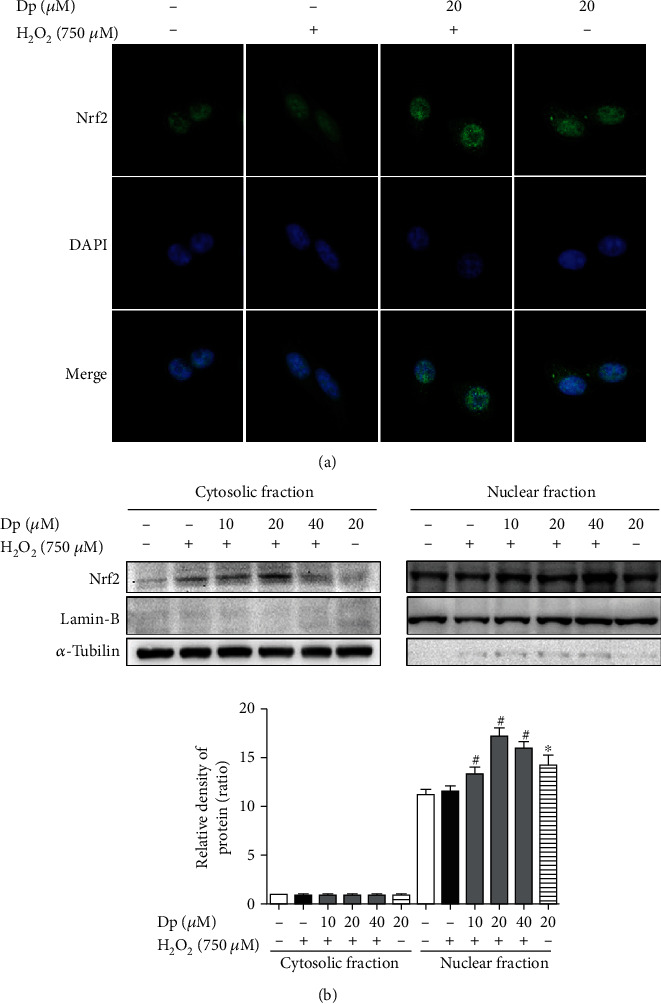
Effects of delphinidin on Nrf2 nuclear translocation in HepG2 cells under H_2_O_2_-induced oxidative stress. (a) Localization of Nrf2 in cells after treating with delphinidin and H_2_O_2_. (b) The expression of Nrf2 protein in cytoplasm and nucleus after treating with delphinidin and H_2_O_2_. *α*-Tubulin was used as a loading control for the total protein. Values were presented as mean ± SD. ^∗^*P* < 0.05 vs. the control group and ^#^*P* < 0.05 vs. the H_2_O_2_-treated group.

To further investigate whether delphinidin could regulate the nuclear translocation of Nrf2, immunofluorescence experiment was performed to locate Nrf2 in HepG2 cells treated with delphinidin (20 *μ*M) and H_2_O_2_ (750 *μ*M). Moreover, Western blot was used to detect the level of Nrf2 in the cytoplasm and nucleus. Compared with the H_2_O_2_-treated group, the green fluorescence expression in the nucleus of HepG2 cells was enhanced after treating with delphinidin (Figure 4(a)). In Figure 4(b), the results showed that few Nrf2 could be detected in the cytoplasm of the control group. Conversely, compared with the H_2_O_2_-treated group, Nrf2 protein expression was increased in the nucleus of the delphinidin intervention group (10 *μ*M, 20 *μ*M, and 40 *μ*M) (*P* < 0.05). These data indicated that delphinidin could promote Nrf2 to enter and accumulate in the nucleus.

### 3.5. Inhibition of Nrf2 Degradation by Delphinidin and H_2_O_2_

**Figure 5:**
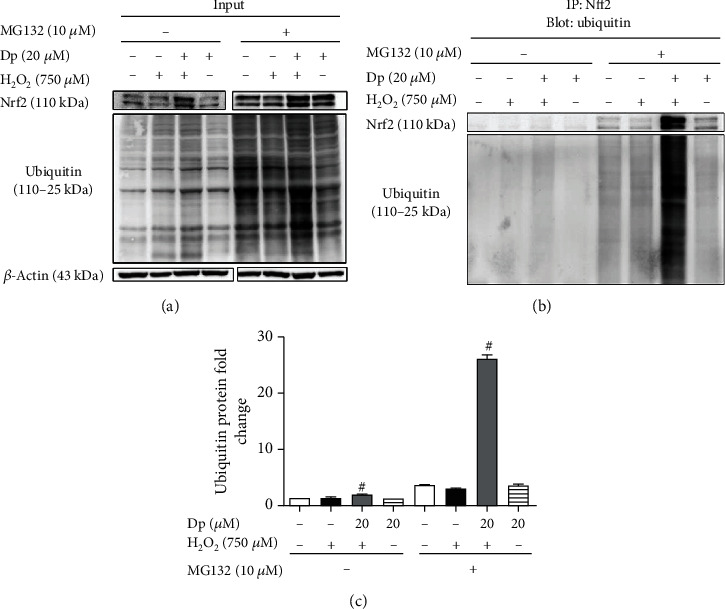
Delphinidin inhibited the degradation of Nrf2 protein in H_2_O_2_-mediated oxidative stress. (a) The total Nrf2 and ubiquitin protein were detected and used as a control. (b) The Nrf2 and ubiquitin protein were detected after immunoprecipitation with Nrf2. (c) The relative protein expression of ubiquitin protein bands. *β*-Actin was used as a loading control for the total protein. Values were presented as mean ± SD. ^#^*P* < 0.05 vs. the H_2_O_2_-treated group.

Nrf2 in the cytoplasm was degraded by the 26S proteasome. The 26S proteasome inhibitor MG132 was employed to pretreat cells and then the ubiquitination of Nrf2 was detected by coimmunoprecipitation and Western blot. After treatment with MG132, the levels of ubiquitinated protein and Nrf2 protein were higher in each group than those without MG132 (Figure 5(a)). In the IP experiment, whether MG132 (10 *μ*M) was added or not, the expression of ubiquitinated Nrf2 was higher in cells supplemented with delphinidin (20 *μ*M) and H_2_O_2_ (750 *μ*M) than other groups (Figure 5(b)). The above results might indicate that delphinidin could attenuate the degradation of Nrf2 by inhibiting the function of the 26S proteasome.

### 3.6. Effect of Delphinidin and H_2_O_2_ on Nrf2 Knockdown Cells

**Figure 6:**
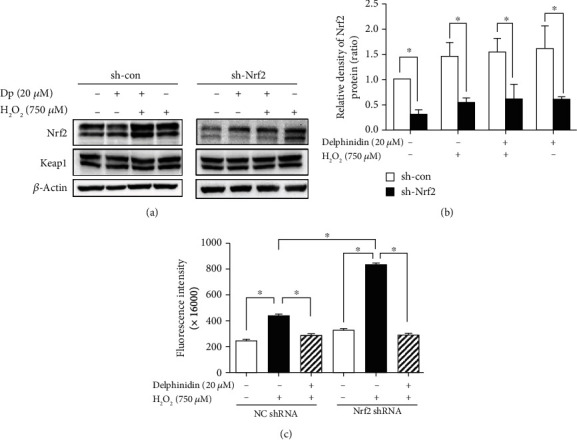
Delphinidin played an important role in Nrf2 elimination of intracellular ROS. The (a) protein bands and (b) relative protein expression of Nrf2 were regulated by delphinidin in Nrf2 knockdown HepG2 cells. (c) Effects of delphinidin (20 *μ*M) on intracellular ROS level induced by H_2_O_2_ in Nrf2 knockdown HepG2 cells. *β*-Actin was used as a loading control for the total protein. Values were presented as mean ± SD. ^∗^*P* < 0.05.

In order to further verify the cytoprotective effect of Nrf2 and delphinidin on oxidative stress, HepG2 cells were transfected with sh-Nrf2 plasmid and subsequently pretreated with 20 *μ*M delphinidin before adding 750 *μ*M H_2_O_2_. The expression of Nrf2 in the control group was significantly reduced after transfecting with sh-Nrf2 plasmid, indicating that the transfection was effective. In addition, the expression of Nrf2 in HepG2 cells treated with delphinidin alone increased in both sh-con and sh-Nrf2 cells, which indicated that delphinidin could upregulate the expression of Nrf2 (Figures 6(a) and 6(b)). Compared with the control group, the results of ROS assay showed that H_2_O_2_ treatment significantly increased the fluorescence intensity of sh-con cells by 0.77 times, while that of sh-Nrf2 cells increased by 1.55 times (*P* < 0.05). Furthermore, the fluorescence intensity of sh-con cells and sh-Nrf2 cells in the delphinidin intervention group was significantly reduced by 32.5% and 64.9%, respectively, compared with that in the H_2_O_2_-treated group (Figure 6(c)). These results indicated that silencing Nrf2 gene enhanced H_2_O_2_-induced oxidative stress in HepG2 cells, while delphinidin reduced oxidative stress back to normal.

## 4. Discussion

According to several reports [29], anthocyanins are water-soluble flavonoids, and their skeleton is a 2-phenylbenzopyran ring structure (Figure 1(a)). They have an antioxidant activity which is associated with the diorthohydroxyl functional moiety. Among various kinds of anthocyanins, delphinidin (Dp) has attracted much attention due to its largest number of hydroxyl groups on the B ring and high antioxidant activity [30, 31]. In this study, the HepG2 cells, which possessed many biological characteristics of hepatocytes and the activity of many phases I and II antioxidant enzymes, were selected to evaluate the antioxidative property effect of delphinidin treatment [32, 33]. So far, there are few reports on the antioxidant activity of delphinidin in HepG2 cells. As a small molecule that easily crosses the cell membrane, H_2_O_2_ is the main component of intracellular ROS produced in many physiological and pathological processes, and it can cause oxidative damage to cells [34, 35]. Therefore, H_2_O_2_ is often used to investigate the mechanism of oxidative stress [34, 36]. In our previous experiments, we treated HepG2 cells with 500 *μ*M H_2_O_2_ and found that although the intracellular ROS level increased, it could not meet the requirements for a stable oxidative stress environment. In this experiment, we selected 750 *μ*M H_2_O_2_ to treat HepG2 cells for 1 h, based on the results of our previous experiments and others' studies [37–39]. In our study, the treatment with 750 *μ*M H_2_O_2_ resulted in decreased cell viability and accumulated ROS in HepG2 cells. Similarly, our study also showed that 750 *μ*M H_2_O_2_ could cause the changes of cells morphology (Figure S1). It was significantly alleviated after pretreatment with delphinidin, which was consistent with the researches of NI [40] and Lee et al. [28]. At present, the toxicity of delphinidin to HepG2 cells has not been studied. In our study, we obtained that the IC50 value of delphinidin was 65.58 *μ*M by using MTT assays (Figure 1(b)). The cells treated with different concentrations (10 *μ*M, 20 *μ*M, and 40 *μ*M) of delphinidin did not cause any damage to the cells (Figure 1(c)). In some other studies, varied concentrations of delphinidin were used to treat with different cells, but it was generally believed that the dose of delphinidin under of 40 *μ*M had no impact to cell proliferation [28, 41]. Compared with the delphinidin intervention group (40 *μ*M), the delphinidin intervention group (20 *μ*M) had a stronger antioxidant capacity (Figures 1(d) and 1(e)). It was suggested that the antioxidant activity of delphinidin in HepG2 cells might be associated with the dose dependence. As we all known, delphinidin has a strong antioxidant capacity, but the molecular mechanism by which delphinidin exerts its antioxidant activity remains elusive.

Polyphenols, such as delphinidin, quercetin, and kaempferol, could exert its cytoprotective properties due to its ability to increase the activity of phase II detoxification enzymes and the ability to directly clear ROS [42–44]. Among multiple phase II detoxification enzymes, HO-1 and NQO1 are considered to produce beneficial responses to oxidative stress in a variety of cells [16, 45]. In fact, that is because heme-derived metabolites produced by HO-1 have powerful antioxidant and cytoprotective activities [46, 47]. In addition, according to the related researches, it has been found that HO-1 and NQO1 could be effectively activated by Nrf2 [30]. In our study, delphinidin treatment significantly upregulated the protein expression of Nrf2 and HO-1 (Figure 3). Unlike the petunidin [48], delphinidin also increased the mRNA level of Nrf2. Moreover, according to several previous reports, we found that polyphenols might activate the Nrf2 pathway by applying slightly oxidative stress [49]. However, in our study, compared with the control group, there were no significant differences in ROS levels when cells were treated with delphinidin (20 *μ*M) alone (Figure 1(e)). Thus, delphinidin did not promote Nrf2 activation in a way that increased slightly ROS levels.

**Figure 7:**
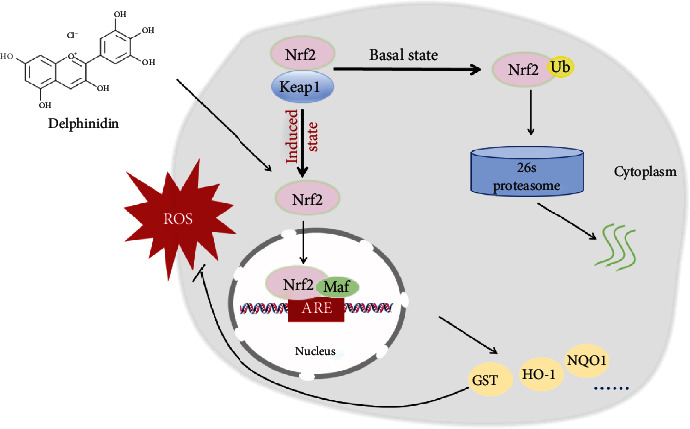
Schematic representation of the process by which Nrf2 exerts its antioxidant effect in cells treated with delphinidin.

In physiological conditions, Nrf2 binds to Keap1 in the cytoplasm and it is continuously ubiquitinated under the synergistic effect of ub-activating enzyme E1, ub-conjugating enzyme E2, and ub-ligating enzyme E3. After that, the ubiquitinated Nrf2 is rapidly degraded by the 26S proteasome. However, if the body is exposed to poisons, drugs, carcinogens, or other electrophiles, Nrf2 will be isolated from Keap1 and transferred into nucleus, where Nrf2 combines with small Maf proteins to form heterodimers before binding to the ARE; finally, the downstream phase II detoxification enzyme is activated [7, 50] (Figure 7). In this study, the immunofluorescence and ubiquitination experiments were used to investigate whether delphinidin exerted an antioxidant effect through this mechanism. The results showed that after delphinidin treatment, the expression of green fluorescence in the nucleus of HepG2 cells was enhanced (Figure 4(a)). In addition, according to the results of Nrf2 protein expression in cytoplasm and nucleus, we could see that delphinidin promoted the nuclear translocation of Nrf2 (Figure 4(b)). Recently, it has been reported that polyphenols such as geraniin [51] and hyperoside [49] could stimulate the expression of Nrf2 in the nucleus, and our results were consistent with these compounds, but different from the quercetin in NB4 leukemia cells [52]. Especially after the pretreatment of MG132, we found that the expression of Nrf2 and ub-Nrf2 was higher in the group which treated with delphinidin (20 *μ*M) and H_2_O_2_ (750 *μ*M) than in other groups (Figure 5). Zhang et al. previously demonstrated that the fisetin, a dietary flavonoid that had the same function as delphinidin in upregulating the expression of Nrf2, prolonged the half-life of Nrf2 protein [2]. These results suggested that delphinidin might reduce the degradation of Nrf2 by inhibiting the function of the 26S proteasome, resulting in a higher expression of the total Nrf2 protein. Actually, Chen et al. previously revealed that the dietary flavonoids with OH groups on the B ring and/or unsaturated C ring could act as proteasome inhibitors [53], which was further supported by our finding. In addition, Qin et al. previously revealed that myricetin [54] and baicalein [17] could inhibit the ubiquitination of Nrf2 and downregulate the expression of Keap1, but this change was not observed after delphinidin intervention. After analyzing the structures of those compounds, we speculated that it might be associated with a 4-carbonyl group on the C ring, which needed further experimental verification. Based on the above results, we considered that delphinidin elevated Nrf2 activation probably by upregulating Nrf2 mRNA expression and promoting Nrf2 nuclear translocation, rather than by inducing Keap1 degradation.

According to the above study, we must consider whether delphinidin could still protect cells from oxidative stress when Nrf2 was knocked down. Although the ROS level of the H_2_O_2_-treated group in the sh-Nrf2 cells was higher than that of the sh-con cells, delphinidin could still decrease the ROS in the sh-Nrf2 cells back to normal level (Figure 6(c)), and we also found that the delphinidin could promote the expression of Nrf2 protein after knocking down Nrf2. The above results indicated the important role of delphinidin in alleviating intracellular oxidative damage in Nrf2 knockdown cells (Figures 6(a) and 6(b)). Considering that there were no significant differences in ROS levels which treated with delphinidin (20 *μ*M) among the sh-con and sh-Nrf2 cells, and the fluorescence intensity of the delphinidin intervention group was lower than that of the control group in sh-Nrf2 cells, we speculated that delphinidin might also play a role in cytoprotective effect by activating other antioxidant pathways, such as phosphatidylinositol-3-kinase (PI3K)/Akt signaling pathway [55] or inhibiting phosphorylation of ERK, JNK, and p38 [56], which needed further experimental verification.

## 5. Conclusion

As an antagonist of the 26S proteasome, delphinidin could upregulate the expression of Nrf2 mRNA, promote the accumulation of Nrf2 in the nucleus, and inhibit the degradation of ub-Nrf2, which activated the expression of the downstream HO-1. In summary, this study demonstrated that delphinidin proposed an antioxidant protective effect of alleviating the toxic effect induced by H_2_O_2_ in HepG2 cells, and the mechanism was through the Nrf2 signaling pathway. It was suggested that delphinidin could be used as a new type of antioxidant to prevent diseases related to oxidative stress.

## Figures and Tables

**Table 1:** The primer sequences for real-time PCR.

Gene	Forward primer	Reverse primer
Nrf2	5′-TACTCCCAGGTTGCCCACA-3′	5′-CATCTACAAACGGGAATGTCTGC-3′
HO-1	5′-CTGACCCATGACACCAAGGAC-3′	5′-AAAGCCCTACAGCAACTGTCG-3′
NQO1	5′-GGCAGAAGAGCACTGATCGTA-3′	5′-TGATGGGATTGAAGTTCATGGC-3′
*β*-Actin	5′-ATCATGTTTGAGACCTTCAACA-3′	5′-CATCTCTTGCTCGAAGTCCA-3′

## Data Availability

The data used to support the findings of this study are available from the corresponding author upon request.

## Supplementary Materials

Supplementary Materials Figure S1: the morphology observation of the different concentrations (10 *μ*M, 20 *μ*M, and 40 *μ*M) of delphinidin with H_2_O_2_ (750 *μ*M)
