# Supplementary material for: Inhibitory Effect of Delphinidin on Oxidative Stress Induced by H2O2 in HepG2 Cells
Source: Oxid Med Cell Longev. 2020 Nov 20;2020:4694760. doi: 10.1155/2020/4694760 (PMC7700032; doi:10.1155/2020/4694760)
Supplement: Supplementary Materials — Figure S1: the morphology observation of the different concentrations (10 μM, 20 μM, and 40 μM) of delphinidin with H2O2 (750 μM) [file 4694760.f1.docx]

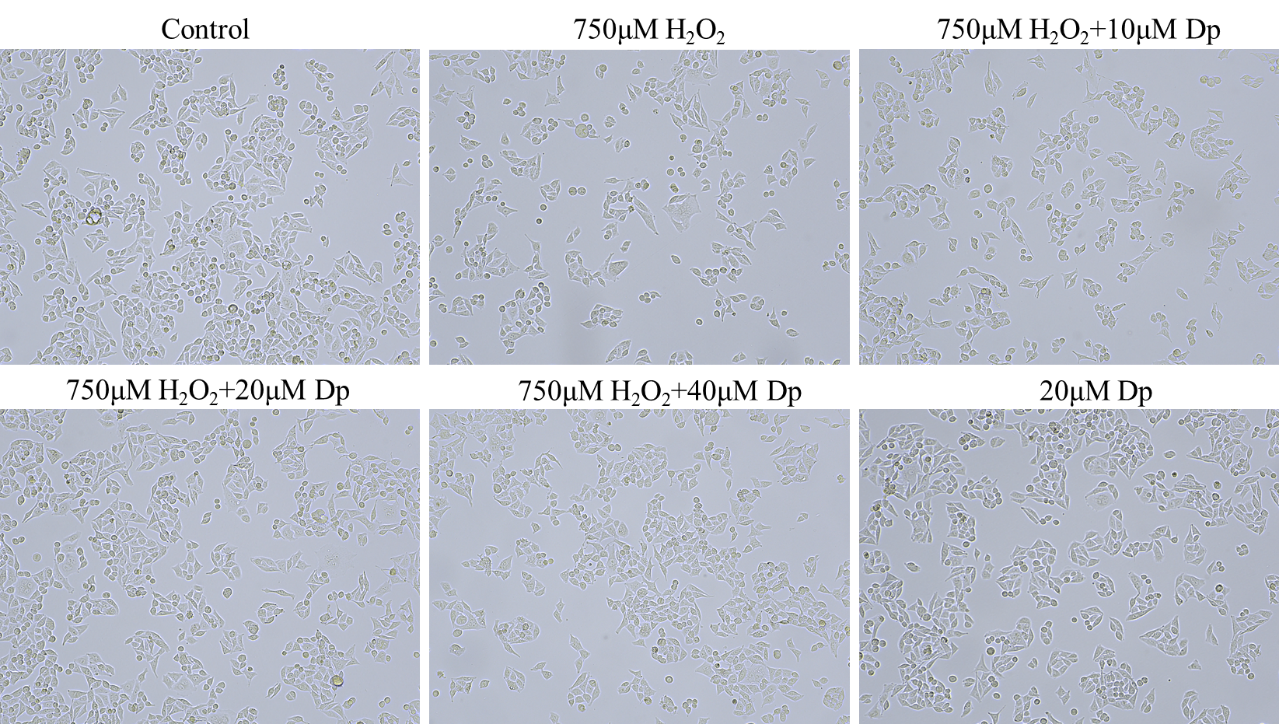


Supplementary Figure 1. The morphology observation of the different concentrations (10 µM, 20 µM, 40 µM) of delphinidin with H_2_O_2_ (750 μM) .Dp: delphinidin.
